# Epigenetic Regulation of IFI44L Expression in Monocytes Affects the Functions of Monocyte-Derived Dendritic Cells in Systemic Lupus Erythematosus

**DOI:** 10.1155/2022/4053038

**Published:** 2022-05-10

**Authors:** Shuaihantian Luo, Ruifang Wu, Qianwen Li, Guiying Zhang

**Affiliations:** Department of Dermatology, Hunan Key Laboratory of Medical Epigenomics, Second Xiangya Hospital of Central South University, Changsha, China

## Abstract

**Background:**

Interferon-inducible 44 like (IFI44L) is a newly discovered interferon-induced gene and has been reported to overexpress in systemic lupus erythematosus (SLE). However, little is known about the mechanism and function of IFI44L overexpression in SLE. In this study, we aimed to investigate the epigenetic mechanism of IFI44L overexpression in SLE monocyte and its potential functions contributing to the pathogenesis of SLE.

**Methods:**

We collected peripheral blood from 20 SLE patients and 20 healthy controls. Expression of IFI44L in monocytes and effects of different signal transducers and activators of transcription (STAT) pathway inhibitors on IFI44L expression were detected. Recruitment of ten-eleven translocation protein (TET) by STAT and methylation of IFI44L promoter were evaluated. Effects of IFI44L overexpression on the expression of surface markers on monocyte-derived dendritic cells (Mo-DCs) were analyzed. T cell differentiation mediated by Mo-DCs and related cytokines production were also analyzed.

**Results:**

Expression level of IFI44L was significantly increased in SLE monocyte. IFI44L expression was decreased most significantly in STAT3 inhibitor compared with other inhibitors. STAT3 regulated IFI44L expression and interacted with TET2 which induced DNA demethylation of IFI44L promoter. Overexpression of IFI44L in monocyte enhanced the maturation and functions of Mo-DC by upregulating costimulatory receptors and inducing Th1/Th17-related cytokines when cocultured with naïve CD4^+^ T cells.

**Conclusion:**

TET2 recruited by STAT3 induces DNA demethylation of IFI44L promoter which promotes IFI44L overexpression in monocyte contributing to the pathogenesis of SLE by enhancing the maturation and functions of Mo-DC. IFI44L is expected to become a new target for treatment of SLE.

## 1. Introduction

Systemic lupus erythematosus (SLE) is a classical autoimmune disease involving many organs and commonly occurs in young and middle-aged women [1, 2]. The interferon system is the main defense of humans against virus and can trigger the expression of more than 20 different interferon-stimulated genes (ISGs) with antiviral and immunostimulatory capacity. Many observations have suggested the critical role of interferon system in SLE and other autoimmune diseases [3–5].

Interferon-inducible 44 like (IFI44L) is a newly discovered ISG and was first identified in the study of the immune responses to viruses. IFI44L has been found to upregulate in the gene profile of many autoimmune diseases besides SLE [6–8]. Our previous studies revealed IFI44L was significantly increased in SLE peripheral blood caused by hypomethylation of IFI44L promoter [9]. There are two significantly different CpG islands located at IFI44L promoter with markedly lower methylation levels in SLE patients than healthy controls [9]. However, epigenetic mechanisms underlying overexpression of IFI44L and potential functions of IFI44L in SLE were still unknown.

Monocyte and dendritic cell (DC) are the main sources of interferon and regulated by the interferon system prominently. Previous studies showed most of ISGs including IFI44L function prominently in mononuclear cell subsets, especially in monocytes and monocyte-derived DCs (Mo-DCs). Signal transducer and activator of transcription (STAT) participated in upstream immunoregulation and epigenetic modifications of many genes in SLE. Ten-eleven translocation protein- (TET-) induced DNA demethylation of specific genes relied on recruitment by upstream transcription factors such as STAT and is involved in differentiation and function of monocytes. Since we have identified overexpression of IFI44L caused by hypomethylation of IFI44L promoter, we supposed upregulation of IFI44L may realize through STAT and TET. Based on our previous studies, we speculated that STAT-mediated activation of TET induced DNA demethylation and upregulated expression of IFI44L in SLE monocyte. In this article, we investigated the epigenetic regulation of IFI44L expression in SLE monocyte and its downstream functions in Mo-DC. The present study explained the mechanism of IFI44L abnormal expression and its critical role in pathogenesis of SLE.

## 2. Methods

### 2.1. Subjects

SLE patients were recruited from the Second Xiangya Hospital of Central South University. All patients fulfilled the 2019 European League Against Rheumatism/American College of Rheumatology Classification Criteria (EULAR/ACR) for SLE [10]. Lupus disease activity was assessed by SLE Disease Activity Index (SLEDAI) at the time of blood collection. Demographics and medications of all patients in this study are listed in Table 1. Healthy controls were recruited from Changsha Blood Center without any autoimmune disease history. This study was approved by the Human Ethics Committee of the Second Xiangya Hospital, Central South University, China. Written informed consent was obtained from all subjects before the study.

### 2.2. Cell Isolation and Culture

Peripheral blood mononuclear cell (PBMC) was isolated from the peripheral blood using density gradient method. CD14^+^ monocytes and naïve CD4^+^ T cells were isolated using human CD14 magnetic beads (Miltenyi Biotec, Germany) and human naïve CD4 T Cell Isolation Kit II (Miltenyi Biotec, Germany) according to the manufacturer's protocol. The isolated cells were cultured in RPMI 1640 medium with 10% fetal bovine serum (FBS) and 1% penicillin/streptomycin. In induced differentiation experiment, Mo-DCs were differentiated from CD14^+^ monocytes using IL-4 (20 ng/mL; Thermo Fisher, USA) and GM-CSF (20 ng/mL; Thermo Fisher, USA) for 7 days, followed by treatment with LPS (20 ng/mL; Sigma, USA) for 3 days. The phenotype of Mo-DC was identified by flow cytometry. In cocultured experiment, naïve CD4^+^ T cells were cocultured with Mo-DCs in a 1 : 5 ratio.

### 2.3. STAT Pathway Inhibitor Treatment

The isolated CD14^+^ monocytes were, respectively, treated with STAT1 inhibitor NSC118218 (10 *μ*mol/L; Selleck Chemicals, USA), STAT3 inhibitor S3I-201 (10 *μ*mol/L; Selleck Chemicals, USA), STAT5 inhibitor MDK6314 (10 *μ*mol/L; Selleck Chemicals, USA), and blank solvent for 2 h. IFN-*α* (1000 U/mL; Sigma, USA) was added, and cells were harvested after 22 hours.

### 2.4. Plasmid and siRNA Transfection

The isolated CD14^+^ monocytes were transfected with overexpression plasmid or small interfering RNA (siRNA) by lentivirus using Gibco CTS LV-MAX Transfection Kit (Thermo Fisher, USA). Briefly, CD14^+^ monocytes were harvested and resuspended in a 500 *μ*L fresh medium. IFI44L-overexpression plasmid pEnter-IFI44L, STAT3-overexpression plasmid, or STAT3-siRNA (Thermo Fisher Scientific, USA) were mixed with CTS™ LV-MAX Transfection Reagent in CTS Opti-MEM I and transfection reagent. The complex was incubated for 10 minutes and then added to cells. Enhancer was added after 16 hours, and cells were harvested after 48 hours.

### 2.5. RT-qPCR

Total RNA was extracted from cells using TRIzol reagent (Thermo Fisher, USA). cDNA was synthesized using PrimeScript® RT reagent kit with gDNA Eraser (TaKaRa, China) according to the manufacturer's protocol. qPCR was performed using SYBR Premix Ex Taq™ (TaKaRa, China) and Roche-LightCycler 96 Real-Time PCR System (Basel, Switzerland). GAPDH was used as an endogenous control. The gene-specific primer sets used in this study are listed in Table 2.

### 2.6. Chromatin Immunoprecipitation (ChIP)

ChIP experiment was performed using ChIP Assay kit (MilliporeSigma, USA). Briefly, DNA and proteins were crosslinked by formaldehyde in buffer containing protease inhibitors. The chromatin was fractured by ultrasonography to interrupt DNA into 500–1000 bp fragments, and supernatants were immunoprecipitated by antibodies. Rabbit anti-STAT3 antibody (Abcam, UK) and control rabbit IgG antibody (Abcam, UK) were used to precipitate the immune complexes. The amount of immunoprecipitated DNA was analyzed by PCR. Primer sets used for IFI44L promoter (*IFI44L-p*) are listed in Table 2.

### 2.7. Coimmunoprecipitation (Co-IP)

Co-IP were performed using Protein G Immunoprecipitation Kit (Thermo Fisher, USA) according to the manufacturer's protocol. Briefly, rabbit anti-pSTAT3 capture antibody (Abcam, UK) and control rabbit polyclonal IgG antibody (Abcam, UK) were used to isolate protein complexes from cell lysate. After being washed and elutioned, the immunoprecipitates were detected by western blot using rabbit anti-pSTAT3 antibody (Abcam, UK) and rabbit anti-TET2 antibody (Abcam, UK).

### 2.8. Bisulfite Pyrosequencing (BSP)

Total DNA was isolated using QIAamp DNA Mini Kit (Qiagen, USA), and bisulfite treatment of DNA was performed using EZ DNA Methylation Kit (Zymo Research, USA) according to the manufacturer's protocol. The 121 bp DNA fragment in IFI44L promoter was amplified by PCR. PCR primers (*IFI44L-p*) are listed in Table 2. The PCR product was sequenced by pyrosequencing with the specific probe using PyroMark Q24 (Qiagen, USA).

### 2.9. Western Blot (WB)

Total proteins were extracted from cell lysates, and protein concentration was analyzed by Bradford Protein Assay Kit (Thermo Fisher, USA). Protein was separated in 8% polyacrylamide gels using sodium dodecyl sulfate-polyacrylamide gel electrophoresis (SDS-PAGE) and then transferred onto polyvinylidene difluoride membranes. The membrane was incubated by rabbit anti-IFI44L (Abcam, UK) and anti-GAPDH (Abcam, UK), followed by anti-rabbit IgG antibody (Abcam, UK). Quantification of immunoreactive bands was normalized to GAPDH by densitometry.

### 2.10. Flow Cytometry

Surface markers was detected by FACSCanto II (BD Biosciences, USA) and analyzed by FlowJo software. Antibodies including anti-human CD40-FITC, CD80-PE, CD83-PE, and CD86-FITC (BD Biosciences, USA) were used for flow cytometry analysis.

### 2.11. Enzyme-Linked Immunosorbent Assays (ELISA)

IFN-*α*, IL-4, IL-17A, and IFN-*γ* in serum or coculture supernatants were detected using Quantikine ELISA Kits (R&D Systems, USA) according to the manufacturer's protocol.

### 2.12. Statistical Analysis

Quantitative data were presented as means ± SEM. Statistical significance was assessed by *t*-test (two-tailed). Correlation analysis was assessed by Spearman's correlation coefficient. All data were calculated in SPSS software.

## 3. Results

### 3.1. IFI44L Expression Is Increased in SLE Monocyte

**Figure 1:**
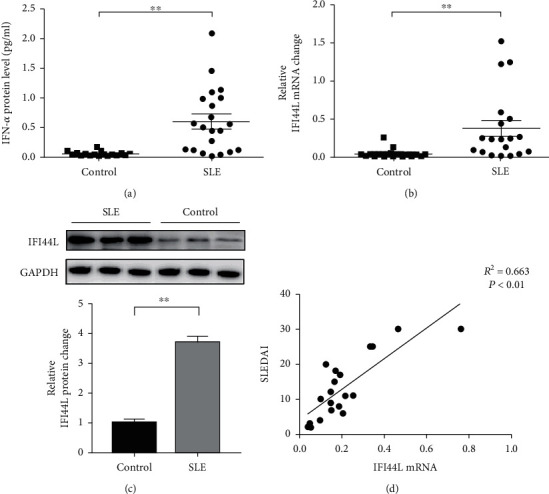
IFI44L is increased in SLE monocyte. The (a) expression level of IFN-*α* in serum and (b) IFI44L mRNA in monocytes from 20 SLE patients and 20 healthy controls were measured by ELISA and RT-qPCR. (c) The expression levels of IFI44L protein in monocytes of 3 representative SLE patients and 3 healthy controls were measured by WB. (d) Correlation between SLEDAI and IFI44L mRNA level. ^∗∗^*P* < 0.01 and ^∗∗∗^*P* < 0.001. Data are shown as mean ± SEMs. Student's *t*-test was used to compare the results. Spearman's correlation coefficient was used for the correlation analysis.

To explore the expression of IFI44L in SLE monocyte, we first detected the expression of IFN-*α* in serum of SLE patients and healthy controls using ELISA. The expression levels of IFI44L mRNA in monocytes of SLE patients and healthy controls were also detected using RT-qPCR. The results revealed both IFN-*α* in serum and IFI44L mRNA in monocytes were significantly increased in SLE patients than healthy controls (Figures 1(a) and 1(b)). The expression levels of IFI44L protein in monocyte were also significantly increased in SLE patients than healthy controls (Figure 1(c)). In addition, we observed the expression levels of IFI44L mRNA in SLE monocytes were positively correlated with the SLEDAI scores (Figure 1(d)).

### 3.2. IFI44L Is Downstream of STAT3 in SLE Monocyte

**Figure 2:**
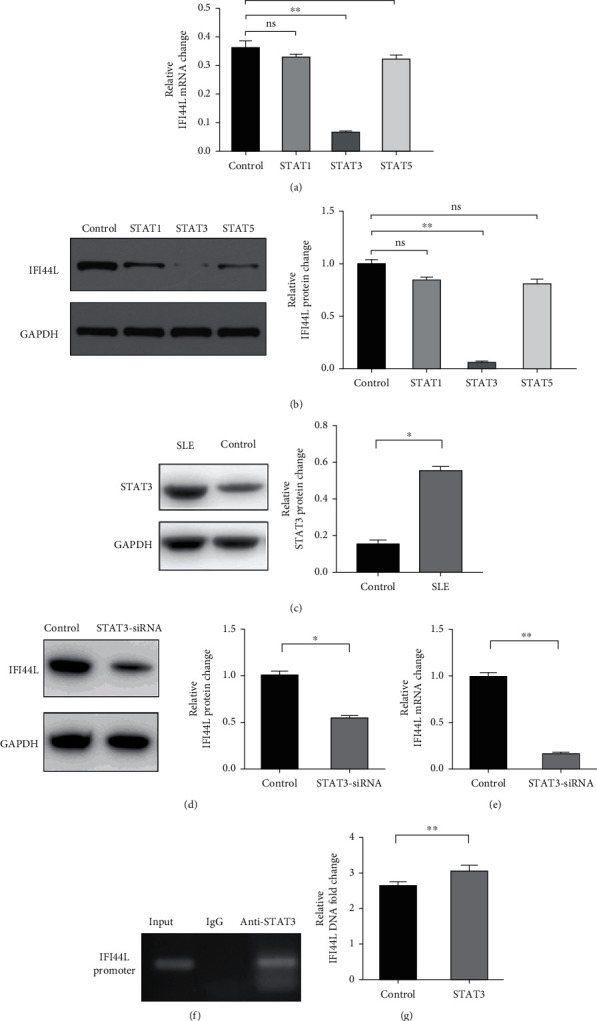
STAT3 is upstream of IFI44L in SLE monocyte. (a, b) RT-qPCR and WB analysis of IFI44L mRNA and protein expression levels in monocytes treated with STAT1, STAT3, and STAT5 pathway inhibitors. (c) WB analysis of STAT3 protein expression levels in monocytes of SLE patient and healthy control. (d, e) RT-qPCR and WB analysis of IFI44L mRNA and protein expression levels in monocytes transfected with STAT3-siRNA or negative control siRNA. (f, g) Interaction between STAT3 and IFI44L promoter was assessed in monocytes by using ChIP-qPCR. ^∗^*P* < 0.05 and ^∗∗^*P* < 0.01. Data are shown as mean ± SEMs. Student's *t*-test was used to compare the results.

To investigate which STAT pathway regulates IFI44L expression, we treated SLE monocytes with STAT1, STAT3, and STAT5 pathway inhibitors and blank solvent, respectively, along with stimulation from IFN-*α*. The results showed the expression level of IFI44L was decreased most significantly in STAT3 inhibitor compared with other inhibitors (Figures 2(a) and 2(b)). To further confirm STAT3 regulated IFI44L expression in monocytes, we compared the expression levels of STAT3 protein in monocytes between SLE patients and healthy controls. As expected, the results showed the expression levels of STAT3 protein in monocytes were significantly increased in SLE patients compared with healthy controls (Figure 2(c)). Additionally, we transfected STAT3-siRNA and negative control siRNA into SLE monocytes, along with stimulation from IFN-*α*. The results revealed the expression levels of IFI44L were significantly decreased in monocytes transfected with STAT3-siRNA compared to negative control siRNA (Figures 2(d) and 2(e)). Moreover, we did ChIP-qPCR assay to confirm the combination of STAT3 and IFI44L in SLE monocytes. As expected, the results showed DNA expression of IFI44L promoter binding to STAT3 was significantly increased compared to the control group (Figures 2(f) and 2(g)).

### 3.3. TET2 Recruited by STAT3 Induces DNA Demethylation of IFI44L in SLE Monocyte

**Figure 3:**
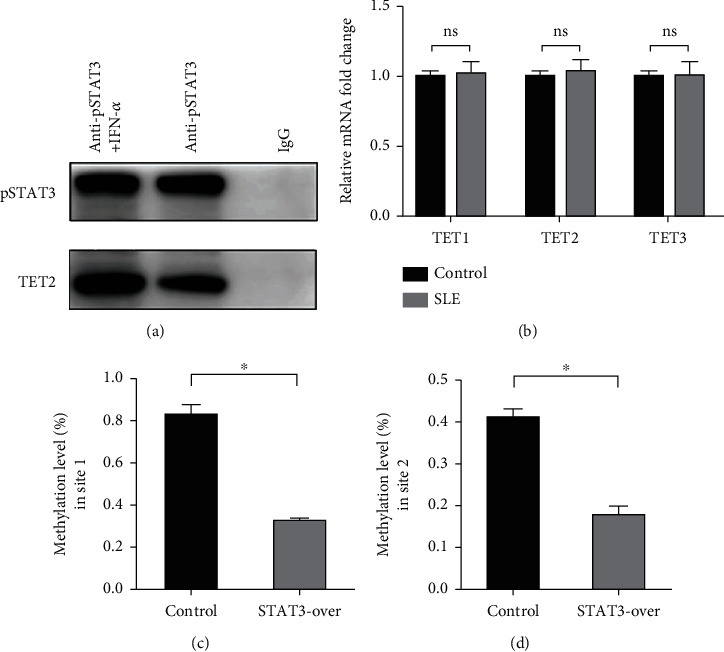
pSTAT3 recruits TET2 inducing DNA demethylation of IFI44L promoter in SLE monocyte. (a) Recruitment of TET2 by IFN-*α*-activated pSTAT3 in CD14^+^ monocytes was detected with Co-IP. Column 1 showed the expression of TET2 recruited by pSTAT3 with IFN-*α* treated. Column 2 showed the expression of TET2 recruited by pSTAT3 without IFN-*α* treated. Column 3 is negative control. (b) RT-qPCR analysis of TET1, TET2, and TET3 mRNA expression levels in CD14^+^ monocytes of SLE patients and healthy controls. (c, d) DNA methylation levels of two CpG sites at the IFI44L promoter in STAT3 overexpression and control groups were detected with BSP. ^∗^*P* < 0.05; ns: not significant. Data are shown as mean ± SEMs. Student's *t*-test was used to compare the results.

To explore TET-induced demethylation of IFI44L dependent on STAT3, we first verified the interaction between phosphorylated STAT3 (pSTAT3) and TET1, TET2, and TET3. As expected, Co-IP results showed a significant interaction between pSTAT3 and TET2 in SLE monocyte (Figure 3(a)). However, there was no significant differences in TET1, TET2, and TET3 mRNA in monocytes between SLE patients and healthy controls (Figure 3(b)). In addition, we transfected STAT3-overexpression plasmid and negative control plasmid into normal monocytes and detected the methylation levels of two CpG sites using BSP. The results showed that DNA methylation levels of IFI44L promoter were significantly decreased in monocytes transfected with STAT3-overexpression plasmid than negative control plasmid (Figures 3(c) and 3(d)).

### 3.4. Overexpression of IFI44L Upregulates Maturation and Costimulatory Receptors of Mo-DC

**Figure 4:**
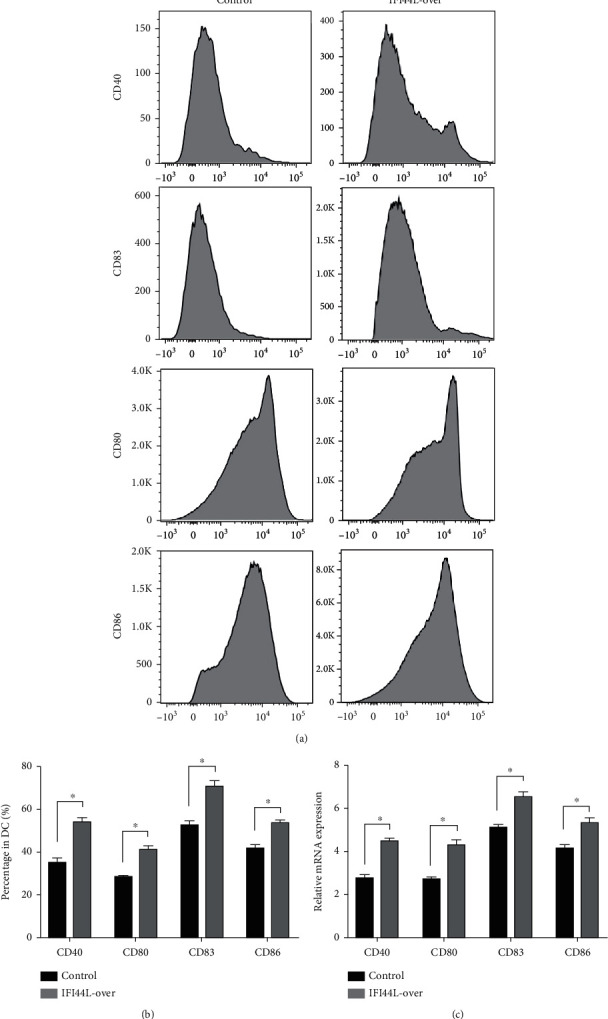
IFI44L overexpression enhances maturation and upregulates costimulatory receptors of Mo-DCs. (a) Flow cytometric analysis of CD40, CD80, CD83, and CD86 expression on Mo-DCs between IFI44L overexpression and negative control. (b) Flow cytometric analysis of CD40, CD80, CD83, and CD86 percentage in Mo-DCs between IFI44L overexpression and negative control. (c) CD40, CD80, CD83, and CD86 mRNA expression levels in Mo-DCs between IFI44L overexpression and negative control. ^∗^*P* < 0.05. Data are shown as mean ± SEMs. Student's *t*-test was used to compare the results.

To explore the functions of IFI44L overexpression in SLE, normal monocytes were isolated and transfected with IFI44L-overexpression plasmid and negative control plasmid and then induced differentiation into Mo-DCs. Flow cytometry showed CD40, CD80, CD83, and CD86 were significantly increased on Mo-DCs transfected with IFI44L-overexpression plasmid compared to negative control plasmid (Figures 4(a) and 4(b)). In addition, RT-qPCR results showed CD40, CD80, CD83, and CD86 mRNA were also significantly increased in Mo-DCs transfected with IFI44L-overexpression plasmid compared to negative control plasmid (Figure 4(c)).

### 3.5. Overexpression of IFI44L in Mo-DC Maintains Upregulation of Th1/Th17-Related Cytokines When Cocultured with Naïve CD4^+^ T Cells

**Figure 5:**
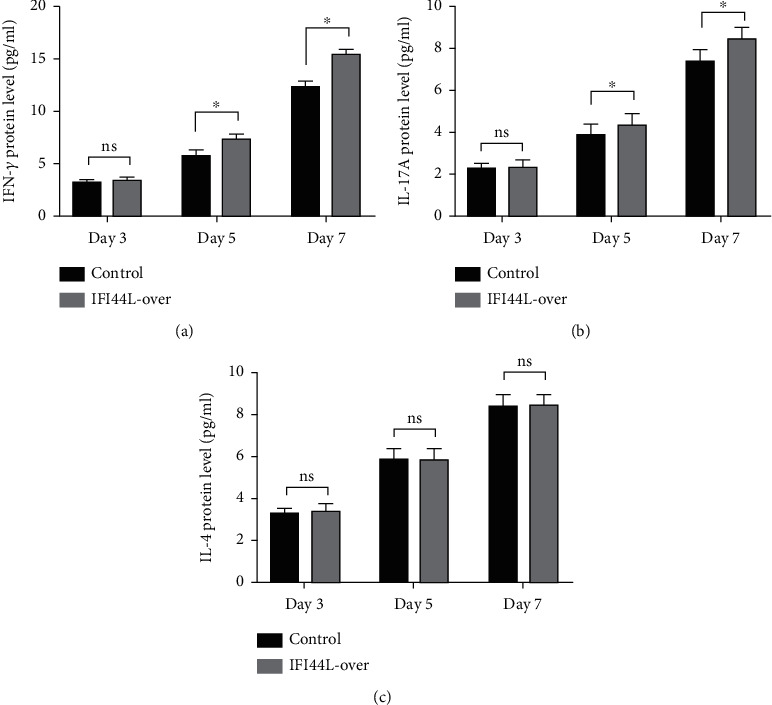
IFI44L overexpression in Mo-DCs maintains upregulation of Th1/Th17-related cytokines when cocultured with naïve CD4^+^ T cells. Protein levels of (a) IFN-*γ*, (b) IL-17A, and (c) IL-4 measured by ELISA in coculture supernatants of naïve CD4^+^ T cells and Mo-DCs transfected with IFI44L-overexpression plasmid or negative control plasmid at day 3, day 5, and day 7. ^∗^*P* < 0.05; ns: not significant. Data are shown as mean ± SEMs. Student's *t*-test was used to compare the results.

To further explore IFI44L-induced upregulation of immune activity and proinflammatory functions of Mo-DC, we did the cocultured experiments. Normal monocytes were isolated and transfected with IFI44L-overexpression plasmid and negative control plasmid and then induced differentiation into DCs. Homologous naïve CD4^+^ T cells were cocultured with transfected Mo-DCs in a 1 : 5 ratio. The results showed IFN-*γ*, IL-4, and IL-17A in the supernatant were significantly increased in both groups at day 7 compared with day 3 (Figures 5(a)–5(c)). The expression levels of IFN-*γ* and IL-17A in the supernatant were significantly increased in the IFI44L-overexpression group than the control group at day 5 and day 7 (Figures 5(a) and 5(b)). By contrast, there was no significant differences of IL-4 expression between two groups at day 3, day 5, and day 7 (Figure 5(c)). Together, our results revealed overexpression of IFI44L maintained upregulation of Th1/Th17-related cytokines and may induce Th1/Th17 polarization in SLE.

## 4. Discussion

Interferon not only plays an important role by itself but also initiates expression of a series of downstream ISGs through intracellular signal transduction [3]. The overexpression of interferon-related genes contributes to an inflammatory environment *in vivo*. Our previous studies were the first to find abnormal expression of IFI44L in SLE peripheral blood and overexpression of IFI44L was most significant in SLE rather than other autoimmune diseases, which provided a new idea for disclosing the pathogenesis of SLE [9]. Monocytes are the cell subsets that express most ISGs in the human body [11], and they are the main inflammation-initiating cells of IFI44L. To clarify the role of IFI44L in SLE, we focused on the monocyte subsets which was consistent with the published studies [7, 8]. In the present study, we found that IFI44L expression was significantly increased in SLE monocytes and positively correlated with the SLEDAI score. SLEDAI score represents disease activity of SLE [12], and the positive correlation between IFI44L expression and SLEDAI score suggests IFI44L may play an important role in aggravating active immune response in the pathogenesis of SLE.

Expression of ISGs requires presence of upstream signaling pathway [13]. STAT pathways are widely linked to immune activation and sustained inflammation in the autoimmune microenvironment [14]. STATs could translocate into the nucleus and bind to IFN regulatory elements which trigger expression of downstream ISGs. In our previous study, we have confirmed that the IL-6/STAT3 pathway participated in upstream immunoregulation and epigenetic modifications of many genes in SLE [15, 16]. STAT4 and STAT6 activated by IFNs have been reported in endothelial and lymphoid cells [17, 18]. In the present study, expression of IFI44L changed significantly when STAT3 has interfered. The results demonstrated that IFI44L was downstream of the STAT3 signaling pathway and that STAT3 was an important regulator of IFI44L overexpression in SLE monocytes.

Abnormal expression of many genes regulated by TET family has been found in many diseases [19–22]. Previous studies have proved that aberrant epigenetic modifications mediated by transcription factors played an important role in the pathogenesis of SLE [15, 23, 24]. TET participated in DNA demethylation involved in monocyte differentiation, and TET-induced demethylation of specific genes relies on recruitment by transcription factors [25]. Here, our data revealed overexpression of IFI44L in SLE was due to the DNA demethylation induced by TET2 which was recruited by STAT3. The epigenetic regulation of the IFI44L gene was confirmed by the significant interaction between STAT3 and TET2 in IP assay and no change of TET protein between SLE and healthy controls. Although this site-specific epigenetic modification cannot be induced by IFN-*α*, this epigenetic regulation may be mediated by transcription factors.

Antigen presentation is the main way that DCs participate in the immune response [26]. Immature DCs could differentiate into mature antigen-presenting DCs with strong costimulatory and T cell activation abilities [27]. This process is associated with high expression of costimulatory receptors on DCs. CD83 is usually known as markers for mature DCs [28, 29], and costimulatory receptors including CD40, CD80, and CD86 also played important roles in DC-initiated T cell proliferation [30]. In the present study, we created an IFI44L-overexpression environment through plasmid transfection into normal monocytes and induced them differentiating into Mo-DCs to simulate the changes in the human body. Our results demonstrated a higher CD40, CD80, CD83, and CD86 expression on Mo-DCs, suggesting an abnormal upregulation of maturation and immune activity in SLE induced by overexpression of IFI44L. The DCs with high levels of costimulatory receptors which stimulated T cell proliferation were associated with high immune responses and proinflammatory effects in SLE patients. These results suggested that IFI44L was a potential proinflammatory factor and partly explained its abilities to promote autoimmune responses.

According to the results of previous studies in coculture of Mo-DCs and autologous naïve CD4^+^ T cells, our results showed IFI44L was also an inducer of different T helper (Th) cell responses [31–34]. We observed that Mo-DCs with IFI44L overexpression enhanced secretion of Th1-related IFN-*γ* and Th17-related IL-17A when cocultured with autologous naïve CD4^+^ T cells and may consequently induce Th1/Th17 polarization. Recent studies have demonstrated that upregulated expression of Th1/Th17-related cytokines and Th1/Th17 immune shift were important pathogenic factors in SLE [35–37]. SLE patients including new-onset cases display high expression of IFN-*γ*/IL-17A in serum and prominent infiltration of Th1/Th17 cells in target organs [38–40]. Our present study revealed that DC-induced Th1/Th17-related cytokines and Th1/Th17 polarization were likely dependent on the IFI44L pathway, which once again suggested IFI44L as a novel initiator of SLE.

In conclusion, TET2 recruited by STAT3 induces DNA demethylation of IFI44L promoter which regulates the expression of IFI44L in SLE monocyte. Overexpression of IFI44L in monocytes enhance maturation and costimulatory receptors of Mo-DCs and maintain upregulation of Th1/Th17-related cytokines when cocultured with naïve CD4^+^ T cells, which all contribute to the pathogenesis of SLE. Our study explained the epigenetic mechanism of IFI44L abnormal expression and revealed overexpression of IFI44L in monocyte participate in the pathogenesis of SLE by aggravating immune activities of Mo-DCs. In the future, we will validate the above mechanisms through larger samples and animal models *in vivo* and explore new methods of SLE treatment by intervening in the expression of IFI44L.

## Figures and Tables

**Table 1:** Demographics and medications of all patients in this study.

Patient no.	Gender	Age (years)	Disease duration	SLEDAI	Treatment regimen
1	F	44	1 year	12	None
2	F	36	2 years	6	Prednisone 20 mg/d
3	F	34	9 months	10	Prednisone 30 mg/d, hydroxychloroquine
4	F	41	8 years	2	Prednisone 12.5 mg/d
5	F	26	5 years	10	Prednisone 30 mg/d
6	F	33	1 year	41	Prednisone 30 mg/d
7	F	53	10 years	8	Prednisone 15 mg/d
8	F	32	17 years	13	Prednisone 30 mg/d
9	F	32	5 years	18	Prednisone 35 mg/d, hydroxychloroquine, cyclophosphamide
10	F	36	3 years	14	Prednisone 10 mg/d, hydroxychloroquine
11	F	32	3 months	14	Prednisone 55 mg/d
12	F	27	3 years	4	Prednisone 10 mg/d
13	F	36	4 years	12	Prednisone 50 mg/d, hydroxychloroquine
14	F	50	10 years	15	None
15	F	50	1 year	12	Prednisone 10 mg/d
16	F	37	6 years	17	Prednisone 15 mg/d
17	F	26	4 months	20	Prednisone 20 mg/d, hydroxychloroquine
18	M	23	1 month	11	None
19	M	36	1 year	30	None
20	M	18	4 years	24	Methylprednisolone 28 mg/d

SLEDAI: Systemic Lupus Erythematosus Disease Activity Index; F: female; M: male; none: no treatment with drugs.

**Table 2:** Gene-specific primer sets used in this study.

Gene name	Primer sequence (5′-3′)
*IFI44L*	Forward: ATGTGACTGGCCAAGCCGTAGT
	Reverse: TGCCCCATCTAGCCCCATAGTGT
*IFI44L-p*	Forward: TGTGGATAGTGATAATTTGTTATAAAGTAA
	Reverse: AACCTCATCCAATCTTAAAACACTTATA
*STAT1*	Forward: CCGTTTTCATGACCTCCTGT
	Reverse: TGAATATTCCCCGACTGAGC
*STAT3*	Forward: GGAGGAGGCATTCGGAAAG
	Reverse: TCGTTGGTGTCACACAGAT
*STAT5*	Forward: GAACACCCGCAATGATTACAGT
	Reverse: ACGGTCTGACCTCTTAATTCGT
*TET1*	Forward: ACCACTTTTGCTACCGACTTG
	Reverse: GGCTGTTCTTTCTGTTCTTGC
*TET2*	Forward: GCAGTGCTAATGCCTAATGG
	Reverse: GAGGTATGCGATGGGTGAGT
*TET3*	Forward: GGACCAGCATAACCTCTACAAT
	Reverse: TCTCCTCGCTACCAAACTCAT
*CD40*	Forward: AGAAGGCTGGCACTGTACGA
	Reverse: CAGTGTTGGAGCCAGGAAGA
*CD80*	Forward: ATGGATTACACAGCGAAGTGGAGAA
	Reverse: AGGCGCAGAGCCATAATCACGAT
*CD83*	Forward: TCCCGGCCCACTTTTTGT
	Reverse: AGGTGGCCCCATGCTACA
*CD86*	Forward: ATGTATCTCAGATGCACTATGGAAC
	Reverse: TTCTCTTTGCCTCTGTATAGCTCGT
*GAPDH*	Forward: ATGGGGAAGGTGAAGGTCG
	Reverse: GGGGTCATTGATGGCAACAATA

## Data Availability

The data that supported the findings of this study are available on reasonable request from the corresponding author.
